# Bactericidal effects of 310 nm ultraviolet light-emitting diode irradiation on oral bacteria

**DOI:** 10.1186/s12903-017-0382-5

**Published:** 2017-06-06

**Authors:** Ayuko Takada, Kenji Matsushita, Satoru Horioka, Yasushi Furuichi, Yasunori Sumi

**Affiliations:** 1 Division of Periodontology and Endodontology Department of Oral Rehabilitation, School of Health Sciences University of Hokkaido, Tobestu, Hokkaido Japan; 2 Department of Oral Disease Research, National Center of Geriatrics and Gerontology, Obu, 747-8511 Aichi Japan; 3 NIIKISO CO., LTD, Tokyo, Japan; 4 Department of Center for Development of Advanced Medicine for Dental Diseases, National Center for Geriatrics and Gerontology, Obu, Aichi Japan

**Keywords:** Phototherapy, Selective toxicity, Reactive oxygen species, *Porphyromonas gingivalis*, Periodontitis

## Abstract

**Background:**

Ultraviolet (UV) light is used for phototherapy in dermatology, and UVB light (around 310 nm) is effective for treatment of psoriasis and atopic dermatitis. In addition, it is known that UVC light (around 265 nm) has a bactericidal effect, but little is known about the bactericidal effect of UVB light. In this study, we examined the bactericidal effects of UVB-light emitting diode (LED) irradiation on oral bacteria to explore the possibility of using a 310 nm UVB-LED irradiation device for treatment of oral infectious diseases.

**Methods:**

We prepared a UVB (310 nm) LED device for intraoral use to examine bactericidal effects on *Streptococcus mutans, Streptococcus sauguinis, Porphyromonas gingivalis,* and *Fusobacterium nucleatum* and also to examine the cytotoxicity to a human oral epithelial cell line (Ca9–22). We also examined the production of nitric oxide and hydrogen peroxide from Ca9–22 cells after irradiation with UVB-LED light.

**Results:**

Irradiation with the 310 nm UVB-LED at 105 mJ/cm^2^ showed 30–50% bactericidal activity to oral bacteria, though 17.1 mJ/cm^2^ irradiation with the 265 nm UVC-LED completely killed the bacteria. Ca9–22 cells were strongly injured by irradiation with the 265 nm UVC-LED but were not harmed by irradiation with the 310 nm UVB-LED. Nitric oxide and hydrogen peroxide were produced by Ca9–22 cells with irradiation using the 310 nm UVB-LED. *P. gingivalis* was killed by applying small amounts of those reactive oxygen species (ROS) in culture, but other bacteria showed low sensitivity to the ROS.

**Conclusions:**

Narrowband UVB-LED irradiation exhibited a weak bactericidal effect on oral bacteria but showed low toxicity to gingival epithelial cells. Its irradiation also induces the production of ROS from oral epithelial cells and may enhance bactericidal activity to specific periodontopathic bacteria. It may be useful as a new adjunctive therapy for periodontitis.

**Electronic supplementary material:**

The online version of this article (doi:10.1186/s12903-017-0382-5) contains supplementary material, which is available to authorized users.

## Background

Ultraviolet (UV) light with a wavelength of 310 nm has been used as phototherapy for various skin diseases such as psoriasis and atopic dermatitis [1–6]. UV light wavelengths are classified as UVC (100〜280 nm), UVB (280〜315 nm) and UVA (315 nm〜400 nm). UVA and UVB radiation has often been used for treatment of skin diseases. Narrowband (NB)-UVB light, the wavelength of which is around 310 nm with a narrow peak, has less side effects for humans than does broadband (BB)-UVB light and has a greater therapeutic effect than the effects of other UV therapies [1, 2]. The safety of NB-UVB light for treatment for skin diseases has also been shown [2, 5]. Unlike PUVA, which is a treatment using both UVA light and psoralen, NB-UVB therapy uses only UV light and thus exhibits no systemic toxicity [1, 2] and also has low cost performance [2]. It has been reported that UVB light irradiation up-regulated regulatory T (Treg) cells [7] and showed immunosuppressive effects in patients with psoriasis and atopic dermatitis. Therefore, NB-UVB light is effective for treatment of various skin diseases that show strong immune responses. NB-UVB might also be useful for treatment of oral mucosal disorders such as periodontitis [8–11], but there has been no report on the use of NB-UVB in dentistry.

It has been shown that UVC light has bactericidal effect [12–14]. The peak of DNA absorption of UV light is 260 nm and its wavelength impairs bacterial DNA by forming pyrimidine (6–4) pyrimidone photoproducts and cyclobutane pyrimidine dimers. The DNA damage also leads to the repression of its transcription and replication and finally induces to cell death [12, 15–17]. UVB light has the same effects to DNA [12, 15], however, there have been few studies on the bactericidal effect of UVB light, especially the effect on oral bacteria.

We hypothesized that UVB would show bactericidal action like UVC, and we produced a small NB-UVB (310 nm) LED device for intraoral use to examine the bactericidal effects on oral bacteria. We also examined the cytotoxicity to human oral epithelial cells to evaluate the safety of the device for oral use. In addition, we examined the production of nitric oxide and hydrogen peroxide from Ca9–22 cells after irradiation with 310 nm UVB-LED with the expectation of an indirect bactericidal effect. This is the first study on the possibility of a narrowband UVB-LED device for intraoral use.

## Methods

### UV-LED irradiation device

**Fig. 1:**
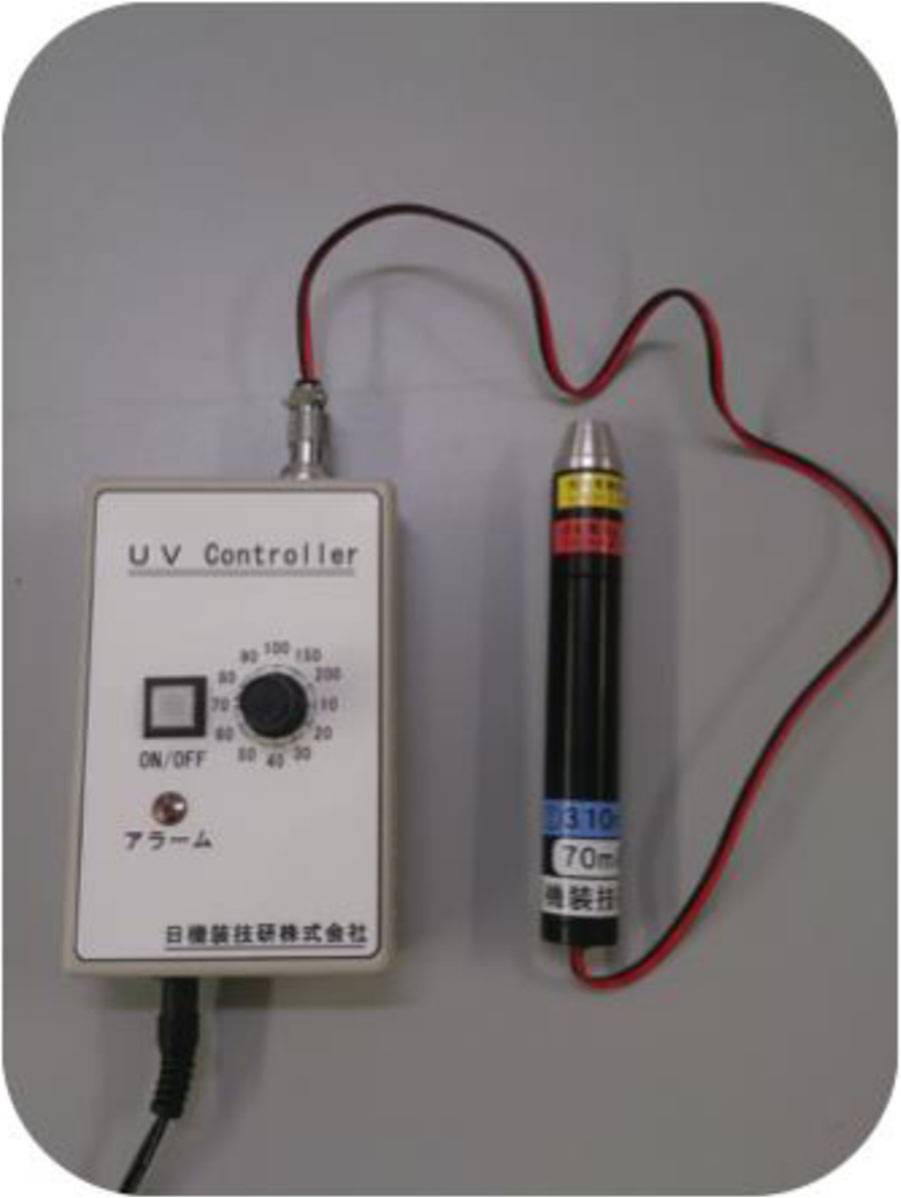
The prototype model of UVB (310 nm) and UVC (265 nm) LED irradiation device

Small UVB (310 nm) and UVC (265 nm) LED irradiation devices were prepared (Fig. 1). The irradiation probe was shaped to a blue LED pen-type light. Power densities of the UVB device and UVC device were 1.75 mW/cm^2^ and 1.77 mW/cm^2^, respectively. Both of them were driven by DC power of the UV control system (10–200 mA and 12 V). The currents of the 310 nm LED and 265 nm LED at a voltage of 12 V were 70 mA and 150 mA (the property of each device being decided by a preliminary experiment by NIKKISO), respectively, for giving an approximate irradiance at the surface of each well in the examinations. The amount of exposure at each radiation time was calculated as E = P × t, where E is energy density (dose) in mJ/cm^2^, P is power density (irradiance) in mW/cm^2^, and t is time in seconds. The data are summarized in Table 1.

**Table 1:** Energy densities of 310 nm and 265 nm by irradiation with ultraviolet light-emitting-diode device. Power fluencies of tested devices were calculated by the following formula: Energy density (mJ/cm^2^) = Power density (mW/cm^2^) × Time (seconds)

Time (sec)	10	30	60	120	180	240
310 nm	17.5	52.5	105	210	315	420
265 nm	17.1	51.3	102.6	205.2	307.8	410.8

Energy density (mJ/cm^2^)

### Bacterial strains and growth media

Brain-Heat Infusion (BHI) agar was purchased from Becton Dickinson and Company (Franklin Lakes, NJ). Modified GAM broth and modified GAM agar were purchased from Nissui Pharmaceutical Co., LTD (Tokyo, Japan). *Porphyromonas gingivalis* ATCC 33277, *Fusobacterium nucleatum* ATCC 25586, *Streptococcus sanguinis* ATCC 10556, and *Streptococcus mutans* ATCC 25175 were purchased from the American Type Culture Collection (Manassas, VA). *P. gingivalis* and *F. nucleatum* were grown on GAM broth and GAM agar, and *S. sanguinis* and *S. mutans* were grown on BHI broth and BHI agar in an anaerobic chamber with Anaeropack Kenki (Mitsubishi Gas Chemical Company, Tokyo, Japan) at 37 °C.

### Oral epithelial cells

A human oral squamous epithelial carcinoma cell line Ca9–22 was obtained from RIKEN Bioresource Center (Ibaraki, Japan). Ca9–22 cells were cultured in Dulbecco’s modified Eagle’s medium (DMEM) (Sigma-Aldrich, St. Louis, MO) containing 10% fetal bovine serum (Hyclone Laboratories, Inc., Logan, UT), 100 U/ml penicillin, and 100 μg/ml streptomycin in a humidified atmosphere with 5% CO_2_ at 37 °C.

### Bactericidal effects

All of the bacteria were grown at 37 °C until the stationary growth phase. The bacterial cells were harvested by centrifugation (8000 × g for 10 min) and re-suspended in sterile phosphate-buffered saline (PBS) to yield a suspension at optical densities (ODs) of 0.4–0.5 at 600 nm with a photospectrometer. Aliquots each of 0.1 ml were used in the experiments. Bacterial suspensions each of 0.1 ml were poured into 96-well plates and were irradiated by the UVB (310 nm)-LED for different times (10, 30, 60, and 120 s). The distance from the light source of UV-LED to bacterial culture (bottom of a 96-wells plate) was 10 mm. The bactericidal effect of UVC (265 nm)-LED irradiation was used as a reference. After irradiation, each of the bacterial suspensions was diluted and seeded on an agar plate. The results were provided by the number of colonies on each agar plate and calculated as CFU/ml.

After incubation for 1–7 days, colonies on the plates were counted. The bactericidal effect was presented as % viability of bacteria calculated by the following formula: %Viability = colony forming unit (CFU)/ml (10, 30, 60, and 120-s irradiations)/ CFU/ml (0-s irradiation) × 100.

### Cytotoxicity to oral epithelial cells

The toxicity of UV-LED irradiation to oral epithelial cells was measured using the Cell Counting Kit-8 (Dojindo, Tokyo, Japan) of WST-8 assay. Briefly, Ca9–22 cells were seeded in 96-well tissue culture plates at a density of 2 × 10^4^ cells/well. The cells were washed three times with phosphate-buffered saline (PBS) and 0.1 ml of PBS was added to each well. The cells were then irradiated by the UV-LED amount of 105, 210, 315, and 420 mJ/cm^2^, that equal irradiation for 60, 120, 180, and 240 s. After UVB light exposure, the supernatants were removed, and fresh DMEM media were added to the wells and the cells were then incubated until desired time points. The activity of dehydrogenase in the supernatants was measured according to the manufacturer's instructions.

### Nitric oxide production in oral epithelial cells

Monolayers of Ca9–22 cells on a chamber slide (Thermo Fisher Science, Waltham, NY) were irradiated by the 310 nm UVB-LED for 60 s. After 24 h, production of nitric oxide in Ca9–22 cells was examined using DAF2-DA (SEKISUI MEDICAL Co., Ltd., Tokyo, Japan).

### Inducible nitric oxide synthase production in oral epithelial cells

A monolayer of Ca9–22 cells on a chamber slide was irradiated by the 310 nm UVB-LED for 60 s and then incubated for 24 h. The cells were washed with PBS three times and were fixed with 4% paraformaldehyde for 10 min. After washing with PBS three times, 0.2% TritonX-100 was added to the chamber for 5 min. After washing with PBS again, the cells were incubated with a blocking buffer (5% serum of goat /0.3% TritonX-100) for 1 h. After washing with PBS three times, the cells were incubated with an anti-iNOS rabbit monoclonal antibody (ab178945: Abcam, Cambridge, UK) at 4 °C overnight. After washing again with PBS three times, the cells were incubated with Alexa Fluor® 488 goat anti-rabbit IgG (H + L) antibody (Life Technologies Japan Inc., Tokyo, Japan) for 1 h. After washing with PBS five times, the nuclei of the cells were stained with DAPI (BioGenex, Fremont, CA). The cells were then observed by a confocal laser scanning microscope (Leica Microsystems, Welzlar, Germany).

### Inducible nitric oxide synthase production in oral epithelial cells (Plymer method)

Immunohistochemical studies were carried out using a polymer staining technique with Histofine Simple Stain MAX-PO(R) (NICHIREI, Tokyo, Japan). A monolayer of Ca9–22 cells on a chamber slide was irradiated by the 310 nm UVB-LED for 60 s and then incubated for 24 h. The cells were washed with PBS three times and fixed with 4% paraformaldehyde for 10 min. After washing with PBS three times, 0.2% TritonX-100 was added to the chamber for 5 min. After washing with PBS again, the cells were incubated with a blocking buffer (5% goat serum/0.3% TritonX-100) for 1 h. After washing with PBS three times, the cells were incubated with an anti-iNOS rabbit monoclonal antibody (ab178945: Abcam, Cambridge, UK) at 4 °C overnight. After washing again with PBS three times, the cells were incubated with a horseradish peroxidase-labeled anti-rabbit immunoglobulin- binding amino acid polymer [Nichirei Simple Stain MAX, PO (R) kit, Nichirei, Co., Tokyo, Japan] for 60 min at room temperature. After washing with PBS five times, sections were incubated in peroxidase substrate solution (ImmPACT DAB Substrate, VECTOR LABORATORIES Inc., CA, USA) until the desired stain was obtained. After washing with PBS five times, the nuclei of cells were stained with hematoxylin. INOS expression was analyzed by NIH-ImageJ macro. The colors of hematoxylin and DAB were digitally separated using Ruifrok and Johnston’s 2 color deconvolution method implemented as NIH-ImageJ macro. Then, iNOS expression was analyzed as the ratio of DAB color, which was calculated by dividing the degree of DAB staining by the number of nuclei.

### Real-time PCR

Ca9-22 cells were irradiated by the 310 nm UVB-LED for 60 s and then incubated for 24 h. Total RNA was extracted from the cells with TRIzol reagent according to the instructions of the manufacturer (Life Technologies). Complementary DNA (cDNA) was synthesized from total RNA using ReverTra Ace -α- (TOYOBO, Osaka, Japan). Real-time PCR was performed using SYBR green Real-time PCR Master Mix (TOYOBO Osaka, Japan) to analyze expression of the iNOS gene (GAPDH being used as a housekeeping gene). The PCR protocol consisted of 40 cycles of denaturation at 95 °C. The set of primers for iNOS was 5′- TCT GCT GGC TTC CTG CTT TC-3′(forward) and 5′-CTG TCC TTC TTC GCC TCG TA-3′(reverse), and the set of primers for GAPDH were 5′-TTT GGT ATC GTG GAA GGA CTC A-3′(forward) and 5′- ATC TCG GGT GTG GTA GGT GA -3′(reverse). RNA expression levels were compared using the ΔΔCt method.

### Hydrogen peroxide production in oral epithelial cells

Hydrogen peroxide production in Ca9–22 cell cultures was measured using the Amplex® Red Hydrogen Peroxide/Peroxidase Assay Kit (Invitrogen, California, USA) according to the manufacturer's instructions. Briefly, a monolayer of Ca9–22 cells in 96-well plates was irradiated by the 310 nm UVB-LED for 60 s. The medium was changed to a reaction mixture in Krebs-Ringer phosphate buffer consisting of 145 mM NaCl, 5.7 mM sodium phosphate, 4.86 mM KCl, 0.54 mM CaCl, 1.22 mM MgSO_4_, 5.5 mM glucose, pH 7.35 and incubated at 37 °C for 0, 1, 3, and 6 h. Fluorescence in the wells was then measured by using a micro plate reader (excitation at 550 nm, emission at 590 nm).

### Bactericidal effect of nitric oxide

DEA NONOate, a nitric oxide donor, was added to 0.1 ml of bacterial suspensions (ODs of 0.4–0.5 at 600 nm in PBS) in 96-well plates. One hour later, the suspensions were diluted and plated on agar plates, and incubated for 1–7 days at 37 °C under anaerobic condition. The colonies on the agar plates were then counted and % viabilities were calculated.

### Bactericidal effect of hydrogen peroxide

Hydrogen peroxide (1 μM and 1 mM) was added to 0.1 ml of bacterial suspensions (ODs of 0.4–0.5 at 600 nm in PBS) in 96-well plates. One hour later, the suspensions were diluted and plated on agar plates, and incubated for 1–7 days at 37 °C under anaerobic condition. The colonies on the agar plates were then counted and % viabilities were calculated.

### Statistical analysis

The data of each examination were expressed as means ± SE and analyzed by one-way analysis of variance (ANOVA). Null hypotheses of no difference were rejected if *p* values were < 0.05.

## Results

### Bactericidal effects of the 310 nm UVB-LED on oral bacteria

**Fig. 2:**
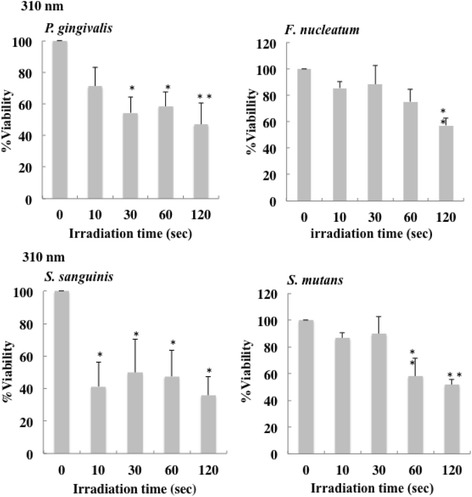
Bactericidal effect of 310 nm UVB-LED irradiation on oral bacteria. Each bacterial suspension (0.1 ml) in 96-well plates was irradiated by a UV-LED. The suspensions were serially diluted and incubated on agar plates at 37 °C anaerobically. Colonies on the plates were counted after incubation for 1–7 days. Bactericidal levels are indicated as viability (%). (*n* = 3 or 5, means ± SE; **P < 0.01 vs. 0 s, *P < 0.05 vs. 0 s)

**Fig. 3:**
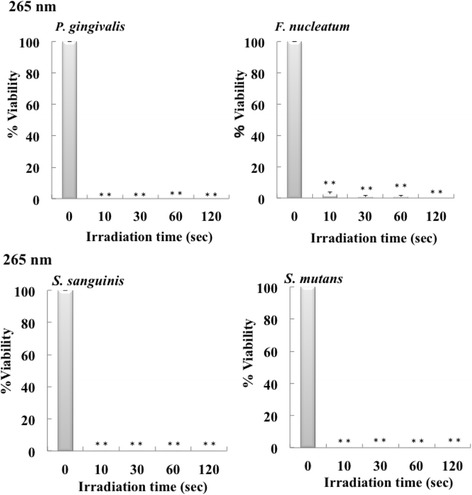
Bactericidal effect of 265 nm UV-LED irradiation on oral bacteria. Each of bacterial suspension (0.1 ml) in 96-well plates was irradiated by a UV-LED. The suspensions were serially diluted and incubated on agar plates at 37 °C anaerobically. Colonies on the plates were counted after incubation for 1–7 days. Bactericidal levels are indicated as viability (%). (*n* = 3, means ± SE; **P < 0.01 vs. 0 s)

The bactericidal effects of irradiation with the 310 nm and 265 nm UV-LEDs on oral bacteria are shown in Figs. 2 and 3. Irradiation with the 310 nm UVB-LED also had bactericidal effects on oral bacteria (Fig. 2), but the activity was lower than that of 265 nm UVC-LED irradiation (Fig. 3). With irradiation by the 310 nm UVB-LED for more than 30 s, viability of *P. gingivalis* was decreased to 47–58% (Fig. 2). The viability of *F. nucleatuim* was reduced to 53.5% by irradiation for 30 s. In addition, viabilities of *S. sanguinis* were decreased to 36–50% by irradiation for more than 10 s, and those of *S. sanguinis* were decreased to 52–58% by irradiation for more than 60 s. On the other hand, irradiation of 265 nm UVC for 10 s killed more that than 97% of these oral bacteria (Fig. 3). We investigated the effect of UVB-LED on biofilms of *P. gingivalis* and *S. mutans*. As shown in Additional file 1, irradiation with the 310 nm UVB-LED also had bactericidal effects on biofilms of *P. gingivalis* and *S. mutans*. With irradiation by the 310 nm UVB-LED for 10–120 s, viability of *S. mutans* was decreased to 69–74%. Viability of *P. gingivalis* was also decreased to 41–54% by the irradiation. A bactericidal effect was also observed by confocal laser scanning microscopy using a LIVE/DEAD BacLight Bacterial Viability Kit (Additional file 2). These results suggest that UVB-LED irradiation is also effective for microbial biofilms.

### Cytotoxicity of 310 nm UVB-LED irradiation

**Fig. 4:**
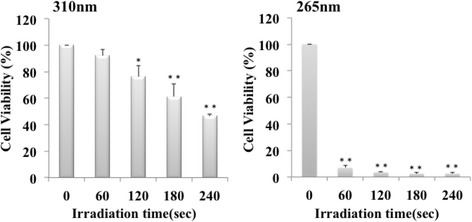
Effect of 310 nm UVB-LED on human gingival epithelial cells. Ca9–22 cells were seeded at a density of 2 × 10^4^ cells/well in 96-well cell culture plates. The cells were irradiated by a UV-LED (265 nm or 310 nm) for 60–240 s and were incubated for 24 h. Cytotoxicity of UV LED irradiation to the cells was measured by WST-8 assay. (*n* = 3, means ± SE; **P < 0.01 vs. 0 s,*P < 0.05 vs. 0 s)

To examine the toxicity of 310 nm UVB irradiation to oral epithelial cells, Ca9–22 cells in culture were irradiated by 310 nm UVB. The results of a WST-8 assay showed that irradiation with 310 nm UVB-LED up to 60 s did not have any cytotoxicity to Ca9–22 cells, though irradiation with the 265 nm UVC-LED for 60 s showed strong cytotoxicity to the cells (Fig. 4). The LD_50_ values of 310 nm and 265 nm were 229.6 s (401.8 mJ/cm^2^) and 32.2 s (57.0 mJ/cm^2^, respectively. These results indicated that 310 nm UV-LED irradiation did not injure oral epithelial cells within 60 s.

### Irradiation of 310 nm UV-LED induces production of ROS in cells

**Fig. 5:**
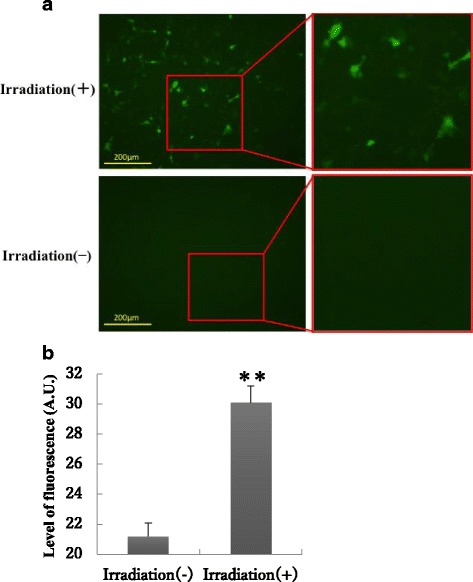
Production of nitric oxide in Ca9–22 cells by irradiation with the 310 nm UVB-LED. **a** Monolayers of Ca9–22 cells were irradiated with 310 nm UVB-LED for 60 s and incubated for 24 h. Nitric oxide production into cells was then examined using DAF2-DA. **b** The level of fluorescence was expressed by using Image J. (*n* = 3, means ± SE; **P < 0.01)

**Fig. 6:**
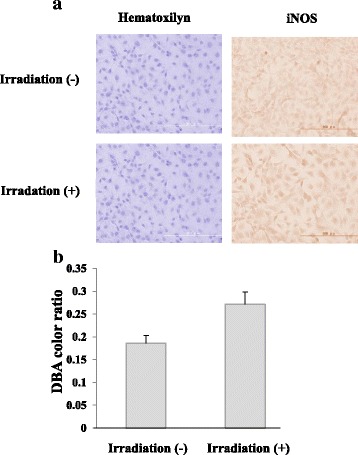
Expression of iNOS in Ca9–22 cells by irradiation with the 310 nm UVB-LED. Monolayers of Ca9–22 cells in a chamber slide were irradiated by the 310 nm UVB-LED for 60 s and incubated for 24 h. **a** Expression of iNOS in the cells was measured by immunofluorescent staining using an anti-iNOS antibody. **b** The levels of DAB color ratio were measured by using Image J. (*n* = 3, means ± SE; *P* = 0.0503)

**Fig. 7:**
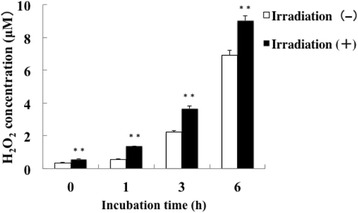
Production of hydrogen peroxide in Ca9–22 cells by irradiation with the 310 nm UVB-LED. Monolayers of Ca9–22 cells were irradiated with 310 nm UVB-LED for 60 s and incubated for 0–6 h. Hydrogen peroxide production into cells was then examined using a kit

UV irradiation induces production of reactive oxygen species (ROS) such as nitric oxide and hydrogen peroxide, and these ROS kill bacteria [18, 19]. Therefore, we examined whether UVB-LED irradiation induces the production of nitric oxide and hydrogen peroxide and whether oral bacteria are killed by these ROS. Production of nitric oxide was observed by 310 nm UVB-LED irradiation for 60 s in Ca9–22 cells (Fig. 5). In addition, production of iNOS was enhanced by irradiation for 60 s in the cells (Fig. 6). In addition, we examined iNOS mRNA expression in UVB-irradiated and non-irradiated cells by RT-PCR. We also observed a significant tendency that iNOS mRNA expression induced in UVB-irradiated Ca9–22 cells was stronger than that in non-irradiated cells (Additional file 3). These results suggest that UVB induced NO production in epithelial cells. Enhanced production of hydrogen oxide was also observed in Ca9–22 cell cultures after irradiation with the 310 nm UVB-LED for 60 s followed by incubation for 1–6 h (Fig. 7).

### ROS decrease the viability of *P. gingivalis*

**Fig. 8:**
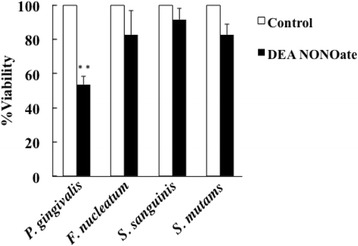
Bactericidal effect of nitric oxide on oral bacteria. Each bacterial suspension (OD of 0.4–0.5 at 600 nm) was incubated with 10 μM DEA NONOate for 1 h. Serially diluted suspensions were seeded on agar plates and incubated for 1–10 days. Colonies on the plates were counted and viability of each bacterium was evaluated. (*n* = 3, means ± SE; **P < 0.01 vs. Control)

**Fig. 9:**
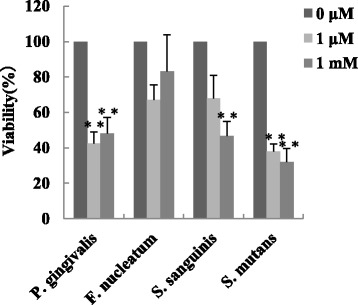
Bactericidal effect of hydrogen peroxide on oral bacteria. H_2_O_2_ (1 μM or 1 mM) was added to each 0.1 ml bacterial suspension (OD of 0.4–0.5 at 600 nm) in 96-well plates and the bacterial suspensions were incubated for 1 h. The serially diluted suspensions were seeded on agar plates, and CFUs were counted. (*n* = 3, means ± SE; **P < 0.01 vs. 0 μM, *P < 0.05 vs. 0 μM)

We next examined the cytocidal effects of these ROS on the tested oral bacteria. Addition of the nitric oxide donor DEA NONOate (10 μM) for 1 h in the cultures killed 56% of *P. gingivalis*, but *F. nucleatum*, *S. sanguinis*, and *S. mutans* were not killed by the treatment (Fig. 8). In addition, the viability of *P. gingivalis* and *S. mutans* was suppressed up to 50% by the addition of 1 μM hydrogen peroxide to the culture, but the viability of *F. nucleatum* and *S. sanguinis* was not suppressed, although 1 mM hydrogen peroxide partially suppressed the growth of *S. sanguinis* and *S. mutans* (Fig. 9).

## Discussion

In this study, we showed that 310 nm UV-LED irradiation had weak bactericidal effects on oral bacteria but showed low toxicity to gingival epithelial cells.

It also induced the production of ROS and it was harmful especially to *P. gingivalis* among the tested bacteria. These results suggest that the 310 nm UV-LED irradiation device may be useful for treatment of periodontitis, which is accompanied by growth of *P. gingivalis* in periodontal pockets.

How does UVB-LED irradiation elicit bactericidal effects on bacteria? UV light, especially UVC light, impairs DNA to induce pyrimidine dimers and then inhibits cell proliferation, elicits apoptosis, and finally induces cell death [20, 21]. UVC light has the strongest bactericidal effects and particularly around 254 nm of UVC is absorbed mostly to DNA. In this study, we observed that 265 nm UVC-LED irradiation has a stronger bactericidal effect than that of UVB-LED irradiation. UVB irradiation also exhibits DNA damage to form pyrimidine dimers, but the effect is very low. Therefore, UVB irradiation may show low toxicity to gingival epithelial cells.

Previous study showed that certain wavelengths have bactericidal effects on oral bacteria [22, 23] For example, 425 nm LED irradiation has a bactericidal effect on *P. gingivalis* [22]. However, the irradiation time in that study was very long and their device was larger than our device, and our device is more suitable for intraoral use. Recently, photodynamic therapy (PDT) has been used for the treatment of various infections by bacteria, fungi, and viruses [24–26]. Red light between 630 and 700 nm had bactericidal effects on periodontopathic bacteria treated with a photosensitizer drug in vitro and in vivo [27]. However, special photosensitizers are needed to obtain a sufficient effect to kill bacteria and there is concern about adverse effects of the sensitizers. However, 310 nm UVB light has been widely used in the field of dermatology and its safety has been well established. Therefore, it is expected that 310 nm UVB light can be safely used even in the oral mucosa. Indeed, our results showed that UVB-LED has a low level of cytotoxicity on oral epithelial cells.

Our results showed that 310 nm UVB irradiation also induced the production of ROS such as nitric oxide and hydrogen peroxide from oral epithelial cells. Periodontopathic bacteria such as *P. gingivalis* are anaerobic bacteria and are sensitive to ROS [28, 29]. On the other hand, other oral resident bacteria such as streptococcci are tolerant to ROS [30, 31]. Therefore, ROS induced by irradiation with a UVB-LED may selectively kill periodontopathic bacteria and might cause a change in oral bacterial flora from periodontopathic to non-periodontopathic. Indeed, *P. gingivalis* was killed by the addition of small amounts of nitric oxide and hydrogen peroxide in vitro. On the other hand, *Streptococcus sanguinis* were not sensitive to those amounts of ROS. Our results also showed that *S. mutans* was sensitive to hydrogen peroxide. Recently, it has been shown that members of the genus Streptococcus strain such as *S. sanguinis* have the ability to produce H_2_O_2_ and inhibit growth of *S. mutans* [32]. Our results follow the relationship between *S. sanguinis* and *S. mutans* [31–33].

Our results also showed that *F. nucleatum,* another periodontopathic bacteria, has no sensitivity to ROS. *F. nucleatum* is sensitive to ROS but has the ability to adapt to ROS and several oral environments [34], and the viability of *F. nucleatum* may therefore not be decreased by irradiation with a UVB-LED.

In this study, we found that UVB irradiation induces the production of nitric oxide and the expression of iNOS in oral epithelium cells. We also found that *P. gingivalis* has strong sensitivity to nitric oxide. It has recently been revealed that nitric oxide has a role in disinfection by breaking down Rieske proteins, which have a critical function for bacteria [35]. A Rieske protein has a bond of iron and sulfur in its molecular structure. When the bond is broken by nitric oxide, the protein loses its function, finally bacteria can not use its protein [35]. *P. gingivalis* produces gingipain, a cysteine protease that is critical for growth of the bacteria. The protease has sulfur bonds in its structure, and the activity of gingipain may be lost by the action of nitric oxide in the same manner as that of Rieske protein, and *P. gingivalis* may therefore exhibit strong sensitivity to nitric oxide. On the other hand, previous study showed that *P. gingivalis* has resistance to excess nitric oxide [36]. Further experiments are needed to understand the killing mechanism.

It has been shown that 310 nm UVB irradiation induces an immunosuppressive reaction in the skin [37]. UVB irradiation causes an increase in IL-10 production and decreases in the number of Th1 cells and increases in the number of Th2 cells [38, 39]. Excess immune reaction is associated with the pathogenesis of chronic periodontitis and relationships among these cytokines, T cells, and periodontitis have been reported [8, 40]. Thus, the immunosuppressive reaction induced by UVB irradiation may be effective for improving chronic periodontitis.

UVB irradiation converts vitamin D from its inactive form to active form in the epithelium [41, 42]. The amount of 1α-hydroxylase, an enzyme that converts vitamin D to its active form, is increased by UVB irradiation in the skin [43]. Furthermore, UVB irradiation induces up-regulation of the expression of vitamin D receptor in the skin [43]. Vitamin D regulates calcium homeostasis and controls bone cell differentiation in alveolar bone. The expression of antimicrobial peptide (AMP) is also increased by UVB irradiation [43, 44]. These findings suggest that UVB light -induced reactions might occur in the oral epithelium and might increase osteogenesis and anti-microbial activities in periodontal tissues.

## Conclusions

Narrowband UVB-LED irradiation has a direct bactericidal effect on oral bacteria but is safe because there was little cytotoxicity to oral epithelium cells. In addition, its irradiation has an indirect bactericidal effect by producing the production of ROS from oral epithelial cells that may kill *P.gingivalis*. It also has promising additional effects such as immune regulation and bone formation. Therefore, narrowband UVB-LED irradiation may be useful as a new therapy for the prevention and treatment of periodontitis.

## Additional files

Additional file 1: Supplemental data #1 Bactericidal effect of 310 nm UVB-LED irradiation on oral bacteria (CFU assay). Suspensions of *P. gingivalis* (0.1 ml, OD of 1.0 at 600 nm) in PBS supplemented with 20 μl human saliva were incubated anaerobically at 37 °C for 24 h. Suspensions of *S.mutans* (0.1 ml, OD of 0.4–0.5 at 600 nm) in BHI broth containing 5% sucrose were also incubated anerobically at 37 °C overnight. These biofilms were irradiated with 310 nm UVB-LED for different periods (10, 30, 60, and 120 s). Colonies on the plates were counted after incubation for 1–7 days. Bactericidal levels are indicated as viability (%). (means ± SE; **P < 0.01 vs. 0 s, *P < 0.05 vs. 0 s). (DOCX 1554 kb)

Additional file 2: Supplemental data #2 Bactericidal effect of 310 nm UVB-LED irradiation on oral bacteria (Microscopical assay). Mixtures of a bacterial suspension (0.2 ml) and saliva (0.1 ml) seeded on an 8-wells chambered coverglass were incubated anaerobically at 37 °C overnight and then irradiated with 310 nm UVB-LED. The biofilms were stained with a LIVE/DEAD BacLight Bacterial Viability Kit and observed by confocal laser scanning microscopy. (DOCX 1581 kb)

Additional file 3: Supplemental data #3 Expression of iNOS mRNA in Ca9–22 cell by irradiation of UVB-LED. Expression level of iNOS mRNA was measured by the RT-PCR. RNA expression levels were compared using the ΔΔCt method. (*n* = 3, means ± SE, *p* = 0.071). (DOCX 30 kb)

## Abbreviations

ANOVA: Analysis of variance
ATCC: American Type Culture Collection
BB: Broadband
BHI: Brain-heart infusion
CFU: Colony forming unit
DAPI: 4', 6-diamidino-2-phenylindole, dihydrochloride
DEA: Diethylamine
DMEM: Dulbecco’s modified Eagle’s medium
iNOS: Inducible nitric oxide synthase
LB: Luria-Bertani
LED: Light emitting diode
NB: Narrowband
OD: Optical density
PBS: Phosphate-buffered saline
PDT: Photodynamic therapy
ROS: Reactive oxgen species
Treg: Regulatory T
UV: Ultraviolet
