# Supplementary figures and images for: Bactericidal effects of 310 nm ultraviolet light-emitting diode irradiation on oral bacteria
Source: BMC Oral Health. 2017 Jun 6;17:96. doi: 10.1186/s12903-017-0382-5 (PMC5461700; doi:10.1186/s12903-017-0382-5)

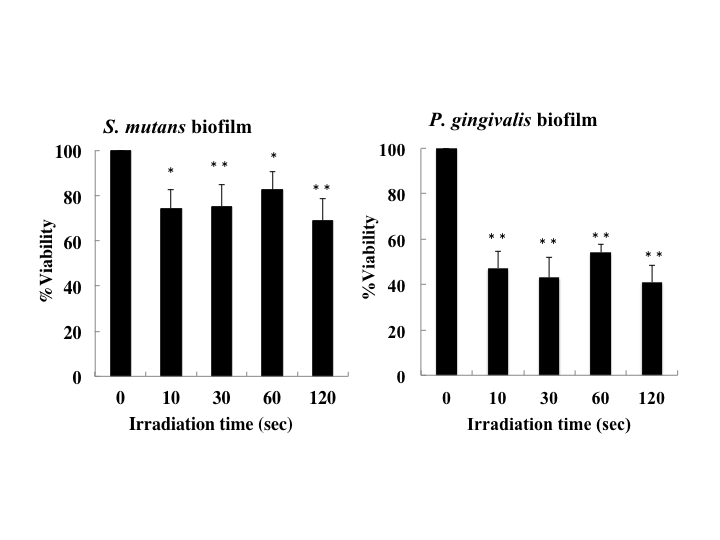


Supplemental data #1

Supplement: Supplementary file 1 — Supplemental data #1 Bactericidal effect of 310 nm UVB-LED irradiation on oral bacteria (CFU assay). Suspensions of P. gingivalis (0.1 ml, OD of 1.0 at 600 nm) in PBS supplemented with 20 μl human saliva were incubated anaerobically at 37 °C for 24 h. Suspensions of S.mutans (0.1 ml, OD of 0.4–0.5 at 600 nm) in BHI broth containing 5% sucrose were also incubated anerobically at 37 °C overnight. These biofilms were irradiated with 310 nm UVB-LED for different periods (10, 30, 60, and 120 s). Colonies on the plates were counted after incubation for 1–7 days. Bactericidal levels are indicated as viability (%). (means ± SE; **P < 0.01 vs. 0 s, *P < 0.05 vs. 0 s). (DOCX 1554 kb) [file 12903_2017_382_MOESM1_ESM.docx]

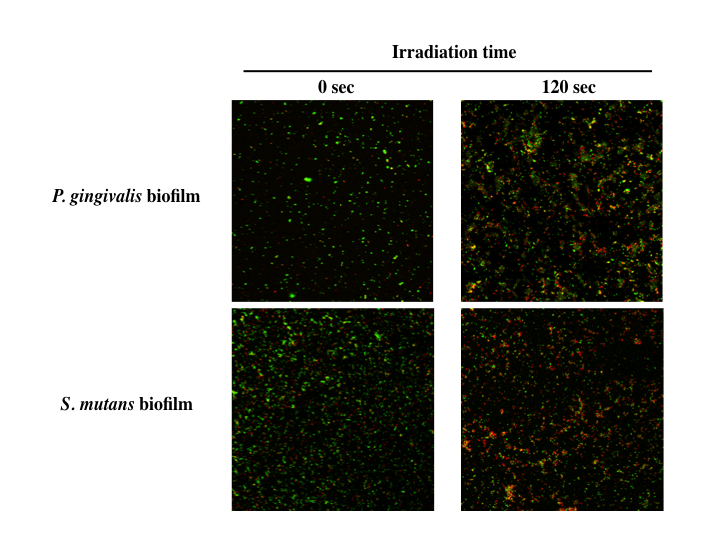


Supplemental data #2

Supplement: Supplementary file 2 — Supplemental data #2 Bactericidal effect of 310 nm UVB-LED irradiation on oral bacteria (Microscopical assay). Mixtures of a bacterial suspension (0.2 ml) and saliva (0.1 ml) seeded on an 8-wells chambered coverglass were incubated anaerobically at 37 °C overnight and then irradiated with 310 nm UVB-LED. The biofilms were stained with a LIVE/DEAD BacLight Bacterial Viability Kit and observed by confocal laser scanning microscopy. (DOCX 1581 kb) [file 12903_2017_382_MOESM2_ESM.docx]
