# Supplementary material for: Bactericidal effects of 310 nm ultraviolet light-emitting diode irradiation on oral bacteria
Source: BMC Oral Health. 2017 Jun 6;17:96. doi: 10.1186/s12903-017-0382-5 (PMC5461700; doi:10.1186/s12903-017-0382-5)
Supplement: Supplementary file 3 — Supplemental data #3 Expression of iNOS mRNA in Ca9–22 cell by irradiation of UVB-LED. Expression level of iNOS mRNA was measured by the RT-PCR. RNA expression levels were compared using the ΔΔCt method. (n = 3, means ± SE, p = 0.071). (DOCX 30 kb) [file 12903_2017_382_MOESM3_ESM.docx]

Supplemental data #3
